# Supplementary material for: The Systems Biology Research Tool: evolvable open-source software
Source: BMC Syst Biol. 2008 Jun 29;2:55. doi: 10.1186/1752-0509-2-55 (PMC2446383; doi:10.1186/1752-0509-2-55)
Supplement: Additional file 1 — SBRT Archive. An archive of the current version of the Systems Biology Research Tool. [file 1752-0509-2-55-S1.zip › sbrt-1.4.0/doc/developers_guide/api/sbrt/kernel/io/ResultsManager.html]

ResultsManager


|  |  |  |
| --- | --- | --- |
| **Overview** \| **Package** \| **Class** \| **Use** \| **Tree** \| **Deprecated** \| **Index** \| **Help** | |  |
| **PREV CLASS**   **NEXT CLASS** | **FRAMES**    **NO FRAMES**     **All Classes** |  |
| SUMMARY: NESTED \| FIELD \| CONSTR \| METHOD | DETAIL: FIELD \| CONSTR \| METHOD |  |


---


## sbrt.kernel.io Interface ResultsManager<T>

**Type Parameters:**: `T` - the type of data an instance of this class can manage.

**All Known Subinterfaces:**: FbaOptOutputFile, MapOutputFile<K,V>, MapTextOutputFile<K,V>, OutputFile<T>

**All Known Implementing Classes:**: AbstractMapOutputFile, AbstractResultsManager, ColumnOrFbaOptOutputFile, ComoFile, ExtremeCurrentManager.Rm, ListResultsManager, MultiOutputFiles, RomoFile, RowOrFbaOptOutputFile, SerFbaOptOutputFile, SerializedOutputFile, SingleRm, SmoFile, TextOutputFile

---

``` public interface ResultsManager<T> ```

This interace is used to represent a results-manager.
A results-manager is supplied with some form of results,
or data, and then it processes those results in an appropriate way. This may
involve writing all, some, or none of the data to a
file or another form of data repository. Other
possibilities exist as well.

**Author:**
:   This interface was written and documented by
    Jeremiah Wright while in the Wagner lab.

---

| **Method Summary** | |
| --- | --- |
| `<S extends T> void` | `addData(S results)`             Adds data to this results manager. |
| `void` | `breakPoint()`             Signals to this manager that a point of discontinuity or change has been reached. |
| `void` | `close()`             Closes this results manager so that no further results can be added. |
| `java.lang.String` | `getName()`             Returns the name of this results manager. |
| `boolean` | `isClosed()`             Indicates if this results manager has already been closed. |

**Method Detail**

### addData

```
<S extends T> void addData(S results)
```

:   Adds data to this results manager.

    :   **Type Parameters:**: `S` - a type extending the primary result type. **Parameters:**: `results` - the results, or data, to be added to this manager. **Throws:**: `java.lang.IllegalStateException` - if `close()` has already been called.

---


### breakPoint

```
void breakPoint()
```

:   Signals to this manager that a point of
    discontinuity or change has been reached.
    The specific actions or response to a call of
    this method is left to implementors of this
    interface.

    :   **Throws:**: `java.lang.IllegalStateException` - if `close()` has already been called.

---


### close

```
void close()
```

:   Closes this results manager so that no further
    results can be added.

---


### isClosed

```
boolean isClosed()
```

:   Indicates if this results manager has already
    been closed.

    :   **Returns:**: `true` if results can no longer be added to this manager; `false` otherwise. **See Also:**: `close()`

---


### getName

```
java.lang.String getName()
```

:   Returns the name of this results manager.

    :   **Returns:**: the name of this results manager, or `null` if no name exists.


---


|  |  |  |
| --- | --- | --- |
| **Overview** \| **Package** \| **Class** \| **Use** \| **Tree** \| **Deprecated** \| **Index** \| **Help** | |  |
| **PREV CLASS**   **NEXT CLASS** | **FRAMES**    **NO FRAMES**     **All Classes** |  |
| SUMMARY: NESTED \| FIELD \| CONSTR \| METHOD | DETAIL: FIELD \| CONSTR \| METHOD |  |


---
